# Akin osteotomy: good staple positioning

**DOI:** 10.1308/003588412X13373405385214t

**Published:** 2012-07

**Authors:** RP Walter, S James, JR Davis

**Affiliations:** South Devon Healthcare NHS Foundation Trust,UK

**Figure 1:**
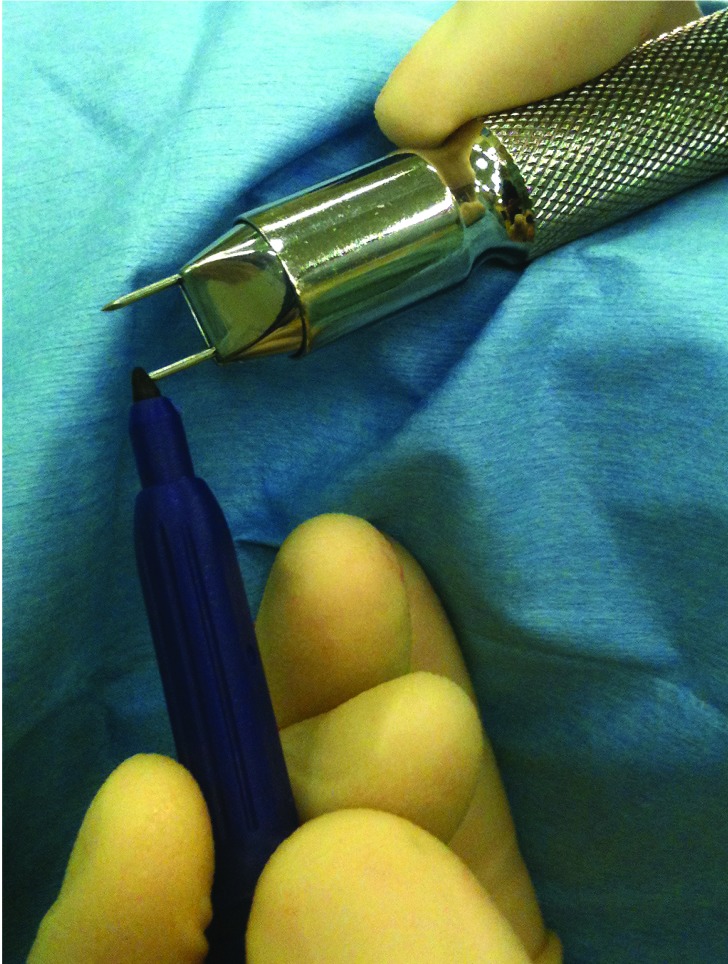
A marker pen is used to apply ink to the staple tip.

**Figure 2:**
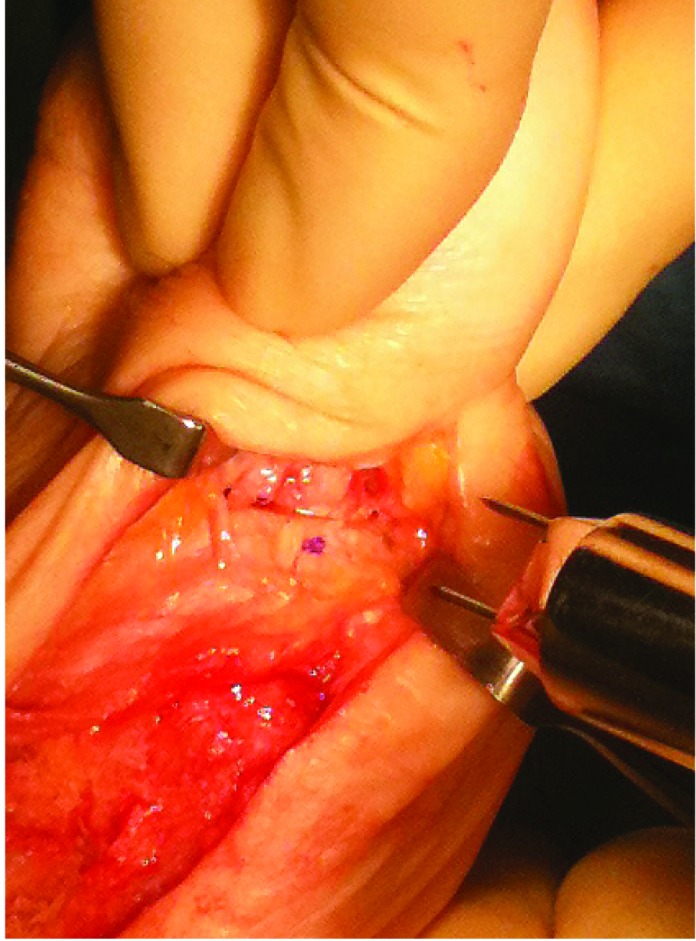
The proximal hole has been marked and drilled ready for staple insertion.

Akin osteotomy is a common component of hallux valgus surgery. Holding the osteotomy in good position with a well placed staple can prove difficult. To aid this, a marker pen is used to apply ink to the tip of the proximal limb of the staple (Fig 1). A hole is drilled in the distal bone and the non-inked staple limb is inserted while the osteotomy is held in the desired position. This causes transfer of ink from staple to bone surface (Fig 2), thereby marking the appropriate site to drill and insert the proximal staple limb.

